# A Clinical and Risk Factor Profile of Patients With Tubo-Ovarian Abscesses: A Retrospective Study From Two District Hospitals in the United Kingdom

**DOI:** 10.7759/cureus.91947

**Published:** 2025-09-09

**Authors:** Rupa Brahma, Richa Kalia, Anwesha Dasghosh

**Affiliations:** 1 Obstetrics and Gynecology, Peterborough City Hospital, Peterborough, GBR; 2 Obstetrics and Gynecology, Manipal Hospital, Kolkata, IND

**Keywords:** c-reactive protein, pelvic inflammatory disease, predictors, recurrence rate, surgical intervention, treatment failure, tubo-ovarian abscess

## Abstract

Introduction: A tubo-ovarian abscess (TOA) represents a severe complication of pelvic inflammatory disease requiring prompt recognition and appropriate management. The decision between medical and surgical intervention remains complex, with various factors influencing treatment outcomes. This study aimed to describe the demographic and clinical characteristics of patients presenting with tubo-ovarian masses and identify predictors for surgical intervention while recording treatment outcomes.

Methods: This retrospective observational study analyzed 69 patients with tubo-ovarian masses treated at two district hospitals in the United Kingdom. All patients initially received broad-spectrum intravenous antibiotics, with surgical intervention reserved for those who failed to respond after 72 hours. Demographic characteristics, clinical parameters, laboratory findings, and treatment outcomes were extracted from electronic medical records. Statistical analysis was performed using Statistical Product and Service Solutions (SPSS, version 25.0; IBM SPSS Statistics for Windows, Armonk, NY), with chi-square tests for categorical variables and Mann-Whitney U tests for continuous data.

Results: The mean age was 42.1±11.1 years, with 39 patients being obese (56.5%). Risk factors included smoking (n=8, 11.6%), intrauterine device (IUD) use (n=12, 17.4%), and diabetes (n=4, 5.8%). Eighteen patients (26.1%) required surgical intervention after failed medical management. Patients requiring surgery had significantly larger abscess size (p=0.036), higher fever prevalence (p=0.045), elevated C-reactive protein (CRP) levels (p=0.004), and longer hospital stays (p<0.001). There were six patients with an early recurrence rate within 45 days (8.7%), with no significant difference between management groups (p=0.647).

Conclusion: Larger abscess size, presence of fever, and elevated CRP levels are significant predictors of surgical intervention in TOA management. The study population demonstrated an older age profile and high obesity prevalence. These findings provide objective clinical indicators to guide treatment decision-making and optimize patient outcomes in TOA management.

## Introduction

Tubo-ovarian abscess (TOA) represents one of the most severe complications of pelvic inflammatory disease (PID), characterised by inflammatory pelvic masses involving the fallopian tubes and ovaries [1]. This condition predominantly affects women of reproductive age and poses significant challenges in clinical management due to its potential for serious morbidity and long-term sequelae, including infertility, increased risk of ectopic pregnancy, and chronic pelvic pain [2,3]. The clinical presentation of TOA typically includes fever, lower abdominal pain, adnexal mass, and elevated inflammatory markers, though the constellation of symptoms can vary considerably [4]. Fever and leukocytosis may sometimes be absent, making diagnosis challenging and potentially delaying appropriate treatment. The condition requires prompt recognition and management to prevent complications such as abscess rupture, sepsis, and reproductive dysfunction [3].

Current management strategies for TOA range from conservative antibiotic therapy to surgical intervention, including laparoscopic or open procedures. Although conservative management of TOAs with antibiotics alone remains first-line, prior literature indicates that better outcomes in the management of TOA were achieved by a minimally invasive approach compared with conservative treatment with antibiotics only. The decision-making process regarding treatment modality remains complex, with various factors influencing the choice between medical and surgical management [5]. Some studies have attempted to identify predictors for treatment failure and the need for surgical intervention in TOA patients [6]. C-reactive protein (CRP) is a sensitive, specific inflammatory marker for predicting TOA in patients with complicated PID, and levels >49.3 mg/L suggest the presence of TOA [7]. Other factors, such as abscess size, patient age, and clinical presentation, have also been investigated as potential predictors of treatment outcomes [6,8].

Despite advances in diagnostic imaging and therapeutic approaches, there remains limited data on the clinical characteristics and treatment outcomes of TOA patients in UK hospital settings. Understanding the demographic profile, risk factors, and predictors of surgical intervention is crucial for optimising patient care and developing evidence-based treatment protocols. Therefore, this study aimed to describe the demographic and clinical characteristics of patients presenting with TOA at two district hospitals in the United Kingdom. The secondary objective was to identify predictors for surgical intervention and record early recurrence rates (within 45 days of initial treatment).

## Materials and methods

This was a retrospective observational study conducted at two district hospitals, Peterborough City Hospital and Hinchingbrooke Hospital, under the North West Anglia NHS Foundation Trust in the United Kingdom, over a defined study period. The study has been reported in accordance with the Strengthening the Reporting of Observational Studies in Epidemiology (STROBE) guidelines for observational studies.

Data source and study population 

Data were obtained from electronic medical records and hospital databases routinely maintained by both institutions. All patients diagnosed with tubo-ovarian masses during the study period were identified through International Classification of Diseases (ICD) coding and cross-referenced with radiology reports confirming the presence of tubo-ovarian collections or abscesses. The diagnosis was established through clinical presentation, laboratory investigations, and radiological imaging (ultrasound, computed tomography, or magnetic resonance imaging). Cases with incomplete medical records or insufficient follow-up data were excluded from the analysis.

Data collection and study variables

The dataset included demographic characteristics (age, BMI); clinical parameters (parity, presenting symptoms including fever); risk factors (smoking status, intrauterine device (IUD) use, diabetes mellitus, history of infertility); radiological findings (size of tubo-ovarian mass, laterality); laboratory parameters (CRP levels on day one of admission); management approach (medical versus surgical); procedural details (interventional radiology drainage, previous surgical history); treatment outcomes (duration of hospital stay); and early recurrence within 45 days of initial treatment.

All patients were initially treated with broad-spectrum intravenous antibiotics as first-line therapy. Failure of conservative management after 72 hours, defined by persistent or worsening clinical symptoms, ongoing fever, or lack of improvement in inflammatory markers, was followed by surgical intervention. Management was classified as medical (antibiotic therapy with or without interventional drainage) or surgical (laparoscopic or open surgical intervention, including drainage, salpingectomy, oophorectomy, or hysterectomy).

Outcome measures

The primary outcome was the demographic and clinical profile of patients with tubo-ovarian masses. Secondary outcomes included identification of predictors for surgical intervention and early recurrence rates within 45 days of initial treatment. Recurrence was defined as re-presentation with clinical and radiological evidence of tubo-ovarian collection requiring further intervention within 45 days of initial discharge.

Ethical considerations

As this study involved retrospective analysis of anonymised clinical data routinely collected for clinical care purposes and no patient identifiers were used, ethical approval was waived.

Data handling and statistical analysis 

Data were extracted using a standardised proforma and compiled in Microsoft Excel 2016 (Microsoft® Corp., Redmond, WA) before analysis using Statistical Product and Service Solutions (SPSS, version 25.0; IBM SPSS Statistics for Windows, Armonk, NY). Descriptive statistics were used to summarise demographic and clinical characteristics. Continuous variables were expressed as mean ± standard deviation for normally distributed data and median with interquartile range for non-normally distributed data. Categorical variables were presented as absolute frequencies and percentages. A comparison between medical and surgical management groups was performed using the chi-square test or Fisher's exact test (when the expected cell count was <5) for categorical variables. For continuous variables, the Mann-Whitney U test was used for non-parametric data comparison. A p-value of <0.05 was considered statistically significant. All statistical tests were two-tailed.

## Results

A total of 69 patients with tubo-ovarian masses were included in this retrospective study. The mean age of participants was 42.1 ± 11.1 years, ranging from 21 to 66 years, with the majority (n=40, 58.0%) being between 21 and 45 years of age (Table 1). More than half of the patients (n=39, 56.5%) were obese with a BMI ≥30 kg/m², while approximately one-fourth were overweight (n=18, 26.1%). Additionally, 45 patients were parous (65.2%) (Table 1).

**Table 1:** Demographic and clinical characteristics of the study participants (n=69) * Mean (SD): 42.1 (11.1) years; median (IQR): 41 (34–51) years

Characteristics	Number (%)
Age*	
21-45 years	40 (58.0)
> 45 years	29 (42.0)
BMI	
Underweight (<18.5 kg/m^2^)	1 (1.4)
Normal range (18.5-24.9 kg/m^2^)	11 (15.9)
Overweight (25.0-29.9 kg/m^2^)	18 (26.1)
Obese (≥30 kg/m^2^)	39 (56.5)
Parity	
Nulliparous	24 (34.8)
Parous	45 (65.2)

Regarding risk factors, eight patients were smokers (11.6%), while 12 had a history of IUD use (17.4%) (Table 2). Diabetes was identified in four cases (5.8%), and infertility was documented in only two patients (2.9%) (Table 2).

**Table 2:** Prevalence of risk factors among the study participants (n=69) IUD, intrauterine device

Risk factors	Number (%)
Smoker	8 (11.6)
IUD use	12 (17.4)
Diabetes	4 (5.8)
Infertility	2 (2.9)

Of the 69 patients, 51 (73.9%) were managed medically, while 18 (26.1%) required surgical intervention. When comparing the two management groups, patients who underwent surgical management had significantly larger tubo-ovarian masses (75.39 ± 22.67 mm vs 64.33 ± 19.00 mm; p=0.036) and were more likely to present with fever (n=20, 66.7% vs n=12, 39.2%; p=0.045) (Table 3). Patients managed surgically also demonstrated significantly higher CRP levels on admission (246.65 ± 117.07 mg/L vs 168.52 ± 83.84 mg/L; p=0.004) and required longer hospital stays (13.0 ± 7.7 days vs 7.5 ± 4.1 days; p<0.001) (Table 3). No significant differences were observed between the groups regarding age, BMI, parity, smoking status, IUD use, diabetes, infertility history, laterality, previous surgical history, or drainage of abscess by interventional radiology (Table 3).

**Table 3:** Comparison of clinical and laboratory parameters on admission between management groups (n=69) IUD, intrauterine device; CRP, C-reactive protein Values are presented as n (%) or mean ± SD; # Based on the chi-square test for categorical data and the Mann-Whitney U test (non-parametric) for continuous data; p<0.05 is considered statistically significant.

Parameters	Medical management (n=51)	Surgical management (n=18)	U-statistic or chi-square statistic	p-value^#^
Age (years)	41.92 ± 11.25	42.56 ± 11.00	0.207	0.837
BMI (kg/m²)	31.33 ± 8.05	30.89 ± 5.85	0.214	0.831
Parity (≥1)	35 (68.6)	10 (55.6)	4.808	0.308
Smoker	4 (7.8)	4 (22.2)	2.684	0.101
IUD use	9 (17.6)	3 (16.7)	0.009	0.925
Diabetes	2 (3.9)	2 (11.1)	1.259	0.262
Infertility	2 (3.9)	0 (0.0)	0.727	0.394
Largest dimension of TO mass (mm)	64.33 ± 19.00	75.39 ± 22.67	2.392	0.036*
Laterality (unilateral)	46 (90.2)	14 (77.8)	1.809	0.179
Fever	20 (39.2)	12 (66.7)	4.031	0.045*
CRP on day 1 (mg/L)	168.52 ± 83.84	246.65 ± 117.07	2.988	0.004*
Previous history of surgery	2 (3.9)	0 (0.0)	0.727	0.394
IR drainage	10 (17.6)	1 (5.6)	1.570	0.274
Days of hospital stay	7.5 ± 4.1	13.0 ± 7.7	3.684	<0.001*

Recurrence within 45 days occurred in six patients (8.7%), with no statistically significant difference between medical and surgical management groups (p=0.647) (Table 4).

**Table 4:** Incidence of early recurrence between management groups (n=69) Values are presented as n (%); # Based on the chi-square test for categorical data; p<0.05 is considered statistically significant.

Recurrence within 45 days	Medical management (n=51)	Surgical management (n=18)	Chi-square statistic	p-value^#^
Yes	4 (7.8)	2 (11.1)	0.179	0.647
No	47 (92.2)	16 (88.9)		

## Discussion

This retrospective study of 69 patients with tubo-ovarian masses from two UK district hospitals provides valuable insights into the demographic profile, clinical characteristics, and management outcomes in a contemporary healthcare setting. The demographic characteristics of our study population demonstrate a notable shift in the traditional epidemiological pattern of TOAs. With a mean age of 42.1 years and 42% of patients being over 45 years, our cohort represents an older population than typically described in classical TOA literature [2,3]. This finding is consistent with emerging trends reported by Halperin et al., who demonstrated that women aged around 45 years are more likely to have larger abscesses with higher inflammatory markers compared to younger women [9]. This age distribution suggests a possible epidemiological shift, potentially reflecting changes in sexual behavior patterns, contraceptive practices, or improved recognition and diagnosis of TOA in older women who may present with atypical symptoms.

The prevalence of obesity in our study population is particularly striking, with 56.5% of patients having a BMI ≥30 kg/m² and only 15.9% having a BMI in the normal range. This finding significantly exceeds the general UK obesity prevalence and suggests obesity may be an important risk factor for TOA development or severity. While our study did not find a significant difference in BMI between medical and surgical management groups (31.33 ± 8.05 vs 30.89 ± 5.85 kg/m²; p=0.831), this may be attributed to the overall high prevalence of obesity across both groups. However, Chan et al. identified BMI ≥24.9 kg/m² as a predictor of failed medical management, suggesting that even moderate overweight status may influence treatment outcomes [6]. The high mean BMI in both our treatment groups (>30 kg/m²) may have masked the predictive value of this parameter, as most patients exceeded the threshold identified by Chan et al. [6]. While obesity has not been extensively studied as a specific risk factor for TOA, it is well-established that obesity can compromise immune function, increase inflammatory responses, and complicate surgical management [10,11]. The high prevalence of obesity in our cohort may also reflect the demographic characteristics of the patient population served by these district hospitals, or could indicate that obese patients are more likely to develop complications requiring hospital admission.

The risk factor profile observed in our study reveals some interesting contrasts with traditional TOA epidemiology. The present study observed a low prevalence of smoking (11.6%) and IUD use (17.4%), although these are identified as more significant risk factors. The low prevalence of diabetes (5.8%) and infertility (2.9%) in our cohort may reflect the older age group and different underlying pathophysiology compared to classical PID-related TOA in younger women.

When examining predictors of surgical intervention, our study identified several clinically significant factors that can guide treatment decision-making. It is important to note that all patients in our cohort initially received broad-spectrum intravenous antibiotics as first-line therapy, with surgical intervention reserved for those who failed to respond after 72 hours of conservative management. The larger mean abscess size in surgically managed patients (75.39 ± 22.67 mm vs 64.33 ± 19.00 mm; p=0.036) aligns with findings from Dewitt et al., who demonstrated that larger abscess size was associated with longer hospitalization and increased complications [8]. This finding is further supported by Chan et al., who identified a specific cutoff of TOA size ≥7.4 cm as a predictor of failed medical management [6]. The mean abscess size of 75.39 mm in the surgically managed group is remarkably close to this threshold, lending strong support to the predictive value of abscess size in treatment decision-making. This size-dependent treatment response is consistent with current clinical guidelines that suggest larger, complex abscesses are less likely to respond to conservative management alone [12,13].

The significantly higher prevalence of fever in surgically managed patients (66.7% vs 39.2%; p=0.045) reflects the more severe inflammatory response and systemic involvement typically associated with larger, more complex abscesses. This finding is consistent with the concept that fever represents a marker of disease severity and systemic inflammatory response, often necessitating more aggressive intervention [14]. The absence of fever in a substantial proportion of medically managed patients (60.8%) supports previous observations that TOA can present without classical inflammatory signs, particularly in older patients or those with chronic, indolent infections [15].

The markedly elevated CRP levels in surgically managed patients (246.65 ± 117.07 vs 168.52 ± 83.84 mg/L; p=0.004) provide strong support for the utility of CRP as a predictor of treatment failure and need for surgical intervention. This finding corroborates the work of Ribak et al., who demonstrated that CRP levels >49.3 mg/L suggest the presence of TOA, and our results extend this by showing that even higher levels may predict the need for surgical management [7]. Akselim et al. [16] reported that CRP on admission >143.5 mg/L had 68.3% sensitivity and 71.1% specificity in predicting the failure of antibiotic treatment. The substantially higher CRP levels in our surgical group suggest that this inflammatory marker could serve as an objective tool for clinical decision-making, particularly when combined with other clinical and radiological parameters.

The significantly longer hospital stay for surgically managed patients (13.0 ± 7.7 vs 7.5 ± 4.1 days; p<0.001) reflects not only the complexity of surgical procedures but also the pre-operative optimization time, post-operative recovery, and the generally more severe disease state in these patients. This finding is consistent with Chan et al., who reported prolonged hospitalization in patients with failed medical treatment (10.8 ± 3.6 vs 4.5 ± 2.0 days; p<0.001) and longer intravenous antibiotic duration (9.4 ± 4.3 vs 3.6 ± 2.2 days; p<0.001) [6]. Their study also noted that patients who failed medical treatment received a mean of 4.0 ± 2.1 days of antibiotics before surgical intervention was deemed necessary, which aligns with our 72-hour observation period. This finding has important implications for healthcare resource planning and patient counseling regarding expected treatment duration.

The 26.1% surgical intervention rate in our study reflects the proportion of patients who failed initial conservative management with intravenous antibiotics after 72 hours. This rate is slightly higher than the 18.4% treatment failure rate reported by Chan et al. [6]. Marshall et al. reported a 35% treatment failure rate when medical treatment failure was defined as requiring surgical intervention beyond 24 hours of antibiotics, demonstrating that treatment failure rates can vary depending on the timeframe used for assessment [17]. The 72-hour interval to assess response to medical treatment, as practiced in the study hospital, represents a more conservative approach compared to the 24-hour threshold used by Marshall et al., potentially contributing to our slightly lower failure rate. The standardized 72-hour trial period for antibiotic therapy provides a clear timeline for clinical decision-making and helps distinguish between patients who will respond to medical treatment versus those requiring surgical intervention. This suggests that the decision for surgical intervention is primarily driven by disease-specific factors (abscess size, inflammatory response, clinical presentation) rather than patient demographic characteristics or predisposing risk factors. This finding emphasizes the importance of individualized treatment approaches based on disease severity rather than patient profile alone.

The recurrence rate of TOA has been reported to be higher when patients are only treated with antibiotics [18]. The early recurrence rate of 8.7% within 45 days observed in our study, with no significant difference between medical and surgical management groups (7.8% vs 11.1%; p=0.647). The similar recurrence rates between groups suggest that when an appropriate treatment modality is selected based on disease characteristics, both medical and surgical approaches can achieve comparable short-term outcomes. Several factors may contribute to TOA recurrence, including incomplete treatment of the initial infection, inadequate drainage of complex multiloculated abscesses, persistent underlying risk factors, and patient non-compliance with antibiotic therapy. Anatomical factors such as the presence of endometriosis, which has been identified as a risk factor for recurrent pelvic inflammatory disease following TOA surgery, may predispose certain patients to repeated episodes [19]. Additionally, immunocompromised states, diabetes mellitus, and continued exposure to sexually transmitted infections can increase the likelihood of recurrence [15]. However, in the present study, the small number of recurrence events limits the power to detect meaningful differences between groups.

Our study has some limitations that warrant consideration. The retrospective design may introduce selection bias and limit the availability of certain clinical data. The relatively small sample size, particularly in the surgical management group, may limit the generalizability of findings and statistical power for detecting smaller differences. Additionally, the study period and specific institutional practices may influence treatment decisions and outcomes. The 45-day follow-up period for assessment of early recurrence, while clinically relevant for acute management, may not capture longer-term outcomes or late complications. Future prospective studies with larger sample sizes and longer follow-up periods are needed to validate these predictive factors and assess long-term outcomes, including fertility preservation, chronic pain, and recurrence rates. Additionally, the development of standardized scoring systems incorporating multiple predictive factors could enhance clinical decision-making and improve treatment outcomes for patients with TOAs.

## Conclusions

This retrospective study of 69 patients with tubo-ovarian masses demonstrates that larger abscess size, presence of fever, and elevated CRP levels are significant predictors of surgical intervention following failed conservative management. The study population exhibited a notably older age profile and high prevalence of obesity compared to traditional TOA cohorts. With a 26.1% surgical intervention rate after 72 hours of antibiotic therapy and an 8.7% recurrence rate within 45 days, outcomes were comparable between medical and surgical management groups when appropriate treatment selection was employed. These findings support the use of abscess size, fever, and CRP levels as objective clinical indicators to guide treatment decision-making and optimize patient outcomes in TOA management.
